# The tripeptide feG regulates the production of intracellular reactive oxygen species by neutrophils

**DOI:** 10.1186/1476-9255-3-9

**Published:** 2006-06-15

**Authors:** Ronald D Mathison, Joseph S Davison

**Affiliations:** 1 Department of Physiology and Biophysics, University of Calgary, 3330 Hospital Drive NW, Calgary, AB, T2N 4N1, Canada

## Abstract

**Background:**

The D-isomeric form of the tripeptide FEG (feG) is a potent anti-inflammatory agent that suppresses type I hypersensitivity (IgE-mediated allergic) reactions in several animal species. One of feG's primary actions is to inhibit leukocyte activation resulting in loss of their adhesive and migratory properties. Since activation of neutrophils is often associated with an increase in respiratory burst with the generation of reactive oxygen species (ROS), we examined the effect of feG on the respiratory burst in neutrophils of antigen-sensitized rats. A role for protein kinase C (PKC) in the actions of feG was evaluated by using selective isoform inhibitors for PKC.

**Results:**

At 18h after antigen (ovalbumin) challenge of sensitized Sprague-Dawley rats a pronounced neutrophilia occurred; a response that was reduced in animals treated with feG (100 μg/kg). With antigen-challenged animals the protein kinase C (PKC) activator, PMA, significantly increased intracellular ROS of circulating neutrophils, as determined by flow cytometry using the fluorescent probe dihydrorhodamine-123. This increase was prevented by treatment with feG at the time of antigen challenge. The inhibitor of PKCδ, rottlerin, which effectively prevented intracellular ROS production by circulating neutrophils of animals receiving a naïve antigen, failed to inhibit PMA-stimulated ROS production if the animals were challenged with antigen. feG treatment, however, re-established the inhibitory effects of the PKCδ inhibitor on intracellular ROS production. The extracellular release of superoxide anion, evaluated by measuring the oxidative reduction of cytochrome C, was neither modified by antigen challenge nor feG treatment. However, hispidin, an inhibitor of PKCβ, inhibited the release of superoxide anion from circulating leukocytes in all groups of animals. feG prevented the increased expression of the β1-integrin CD49d on the circulating neutrophils elicited by antigen challenge.

**Conclusion:**

feG reduces the capacity of circulating neutrophils to generate intracellular ROS consequent to an allergic reaction by preventing the deregulation of PKCδ. This action of feG may be related to the reduction in antigen-induced up-regulation of CD49d expression on circulating neutrophils.

## Background

Through the release of proteins and peptides the salivary glands are active participants in the digestion and in the maintenance of the health and integrity of the oral and gastric mucosa [1]. Less well recognized is the role of salivary endocrine factors in the modulation of systemic immune and inflammatory reactions [2,3]. One of these endocrine factors is the seven amino acid peptide – submandibular gland peptide-T (SGP-T; sequence = TDIFEGG), which markedly attenuates the severity of anaphylactic and endotoxic reactions [4,5]. This heptapeptide can be truncated to a biologically active tripeptide (FEG) which, when converted to its D-isomeric form (feG), produces a significant reduction in type I hypersensitivity (allergic) reactions of the intestine, heart, skin and lungs [6-10].

Traditionally allergic reactions are often associated with eosinophil activation and infiltration into the airways [11], even when the reaction occurs outside the lungs in peripheral tissues such as the intestine [12] or the skin [13]. However, 50% of asthma cases are non-eosinophilic in nature and attributable to neutrophilic airway inflammation, possibly triggered by bacterial endotoxin, particulate and gaseous air pollution, viral infection, and allergens or their mediators [14], and a significant neutrophil component is recognized with allergic rhinitis [15], and the vascular permeability changes elicited by intestinal allergy [10]. With the Sprague-Dawley strain of rat airway allergic reactions shows a large neutrophilic inflammation [16], whereas with the Brown Norway strain influxes of neutrophils, eosinophils and lymphocytes occur [6]. Treatment with feG reduces this influx of leukocytes in antigen-challenged Brown Norway rats [6], and the peptide is also potent inhibitor of human and rat neutrophil adhesion and migration [10,17,18].

The primary role of the neutrophil in the inflammatory response is to seek, bind, ingest and destroy invading pathogens, although the neutrophil is also activated by allergic reactions. Since activation of neutrophils is associated with an increase in respiratory burst with the generation of ROS, an expectation is that feG, as a potent suppressor of several neutrophil functions, would also regulate the respiratory burst in neutrophils. In this study we report that feG suppresses the increase in intracellular ROS production by circulating neutrophils elicited by a type I hypersensitivity reaction.

## Methods

### Animals and sensitization

The University of Calgary Animal Care Committee approved the research protocol, which conforms to the guidelines of the Canadian Council on Animal Care. Sprague-Dawley rats (Charles River Canada, Saint-Constant, QC), with an initial weight of 160–175 g were sensitized with an intraperitoneal injection of 1 mg OA and 50 ng pertussis toxin (Sigma Chemical, St. Louis, Mo.) as an adjuvant [4,19]. Four to six weeks following sensitization the animals, now weighing 300–350 g, were divided into four groups and treated as follows 18 hours before collection of the white blood cells: (1) 100 mg/kg of naïve antigen (BSA) into the stomach by gavage (BSA group; n = 25); (2) 100 μg/kg of feG intraperitoneally, and 100 mg/kg of BSA (feG group; n = 25); (3) 100 mg/kg of sensitizing antigen into the stomach by gavage (OA group; n = 25); or (4) 100 μg/kg of feG intraperitoneally, and 100 mg/kg of OA (OA+feG group; n = 25). A dose of 100 μg/kg of feG was used as it provides maximal inhibition of intestinal allergic reactions in sensitized rats [20].

### Leukocyte preparation

Under halothane anaesthesia 9–10 mL of blood was collected by cardiac puncture into a 10 mL syringe, containing 1 ml of 3.8% Na citrate, an anticoagulant. The blood was diluted with polymorphonuclear leukocyte (PMN) buffer without calcium in a 50 mL polypropylene centrifuge tube, and centrifuged at 400 g for 15 min at 4°C. The PMN buffer was of the following composition: 138 mM NaCl, 2.7 mM KCl, 3.2 mM Na_2_HPO_4_·12H_2_O, 5.5 mM glucose. The white blood cells were removed from the surface of the pellet with a plastic Pasteur pipette, and contaminating red blood cells were lysed with 4 volumes of 0.15 M NH_4_Cl for 10 min at room temperature. The volume of the polypropylene centrifuge tube was completed to 50 mL with PMN buffer without calcium, and after a second spin at 400 g for 10 min at 4°C, the supernatant was discarded. The pellet was washed with calcium free PMN buffer and centrifuged again 400 g for 10 min at 20°C. The supernatant was discarded and the cells resuspended in 1 mL of PMN buffer containing calcium (1.2 mM CaCl_2_), and stored on ice until used.

Total blood leukocyte counts were determined with a Hylite haemocytometer (Hauser Scientific, Boulder, CO) using Trypan Blue exclusion as a marker of cell viability. From FACS analysis (see below) the percent of neutrophils in the blood samples was determined.

### Measurement of intracellular ROS

A fluorescent probe and flow cytometry techniques provide a rapid and sensitive method for measuring intracellular ROS generation. The fluorescent probe, DHR, (Sigma-Aldrich) is specifically responsive to H_2_O_2 _accumulation [21], which is generated by the myeloperoxidase in neutrophil granules.

Leukocytes (1 × 106/ml) were preincubated, with continuous shaking, for 15 min at 37°C in PMN-Ca^2+ ^buffer, containing 0.25 μmol/l DHR. The cells were then stimulated with different concentrations of PMA (10^-8 ^to 10^-5^M) for 10 min at 37 °C, and then stored on ice to stop reactions until flow cytometry analysis. The results are expressed as the mean fluorescence intensity (MFI).

To evaluate the role of PKC in the production of intracellular ROS leukocytes (1 × 106/ml) were preincubated, in the presence of DHR, for 15 min at 37°C with one of several PKC inhibitors – Gö6976 (EMD Biosciences, San Diego, CA); hispidin (Sigma-Aldrich, St. Louis. MO) and rottlerin (ALEXIS Biochemicals, San Diego, CA). The PKC inhibitors, which show some isoform specificity, were used at the IC50 values identified using isolated enzymes and whole cells (Table 1).

**Table 1:** Protein kinase C inhibitors, their specificity and IC50 values.

**Inhibitor (Selectivity)**	**IC50 Values**	**Concentration Used**	**Reference**
Gö6976 (α > β)	PKCα = 2 nM; PKCβ1 = 6 nM	3 nM	[66, 67]
Hispidin (β)	PKCβ = 3 μM	6 μM	[68]
Rottlerin (δ)	PKCδ = 2–6μ M; PKCα,β,γ = 30–40 μM	6 μM	[51, 69]

### Cell staining for CD11b/c and CD49d

One million cells were incubated with flourescein-conjugated antibody for 30 min at 4°C in the dark in polypropylene tubes. Rat anti-CD49d monoclonal antibody (CD49d:FITC; clone TA-2) was from Serotec Inc. (Raleigh NC, USA), and mouse anti-CD11b/c monoclonal antibody, (CD11b/c:FITC; clone OX 42) was from Abcam, Inc. (Cambridge MA, USA). Following incubation with the antibody 1 mL of cold PBS was added and the cells centrifuged at 400 g for 10 min at 4°C. The supernatant was decanted and 500 μL of PMN buffer was added to the cells, which were then aspirated with a plastic Pasteur pipette to a polystyrene tube for reading with a Fluorescence Activated Cell Sorter. The effects of the peptides on the binding of antibodies to cell surface molecules were evaluated by determining the mean fluorescence intensity (MFI) of cells after subtracting the background.

### Flow cytometry

Analyses of fluorescence were carried out on a Becton Dickinson (BD) FACSVantage SE™ System at the Flow Cytometry Core Facility at the University of Calgary. With the FACS leukocytes are distinguished and neutrophils readily identified by forward/side light scatter, which represent cell size and granularity, respectively. In all 10^4^events are collected in each gate, and the fluorescence recorded under 488 nm excitation. Green fluorescence from DHR was measured in the FL1 channel through a 525 nm band-pass filter (BP) in combination with a 550 nm dichroic long pass (DL) mirror. Fluorescence emissions are recorded using photomultiplier gain settings. ROS production was quantified by mean fluorescence intensities (MFI).

### Release of superoxide anion

Neutrophils (10^6^) were suspended in PMN buffer containing cytochrome C (1 mg/ml; Sigma-Aldrich) and incubated at 37°C. Each sample was read at 550 nM along with a reference sample containing 1440 units of superoxide dismutase (Sigma-Aldrich) in a dual-beam spectrophotometer (Hitachi, U200 spectrophotometer). The rate of superoxide production in response to 10^-5^M PMA was calculated from the slope of the line [22], and was expressed as μmol superoxide/10^6 ^neutrophils. The percent neutrophils was determined by flow cytometry, and was based on total leukocyte counts, determined with a Hylite haemocytometer (Hauser Scientific, Boulder, CO) using Trypan Blue exclusion as a marker of cell viability, the number of neutrophils were calculated.

To evaluate the role of PKC on the release of superoxide leukocytes (1 × 106/ml) were preincubated for 5 min at 37°C with one of several PKC inhibitors (Gö6976/PKCα; hispidin/PKCβ; rottlerin/PKCδ) during a 5 min preincubation period. The results were analyzed by one-way analysis of variance (ANOVA) for differences between animal groups (BSA, feG, OA and OA+feG) with a specific PKC inhibitor (Gö6976/PKCα; hispidin/PKCβ; rottlerin/PKCδ) and for differences between the PKC inhibitors for a specific animal group.

### Data analysis

The results are presented as the mean ± SEM. The statistical functions used that associated with Excel (Microsoft Office XP, Redmond, WA). Comparisons between two groups were made using the unpaired Student's t-test. Where appropriate one-way analysis of variance was applied using a Student's t-test for post hoc analysis. Statistical values reaching probabilities of p < 0.05 were considered significant.

## Results

### Leukocyte numbers and percent neutrophils

**Figure 1:**
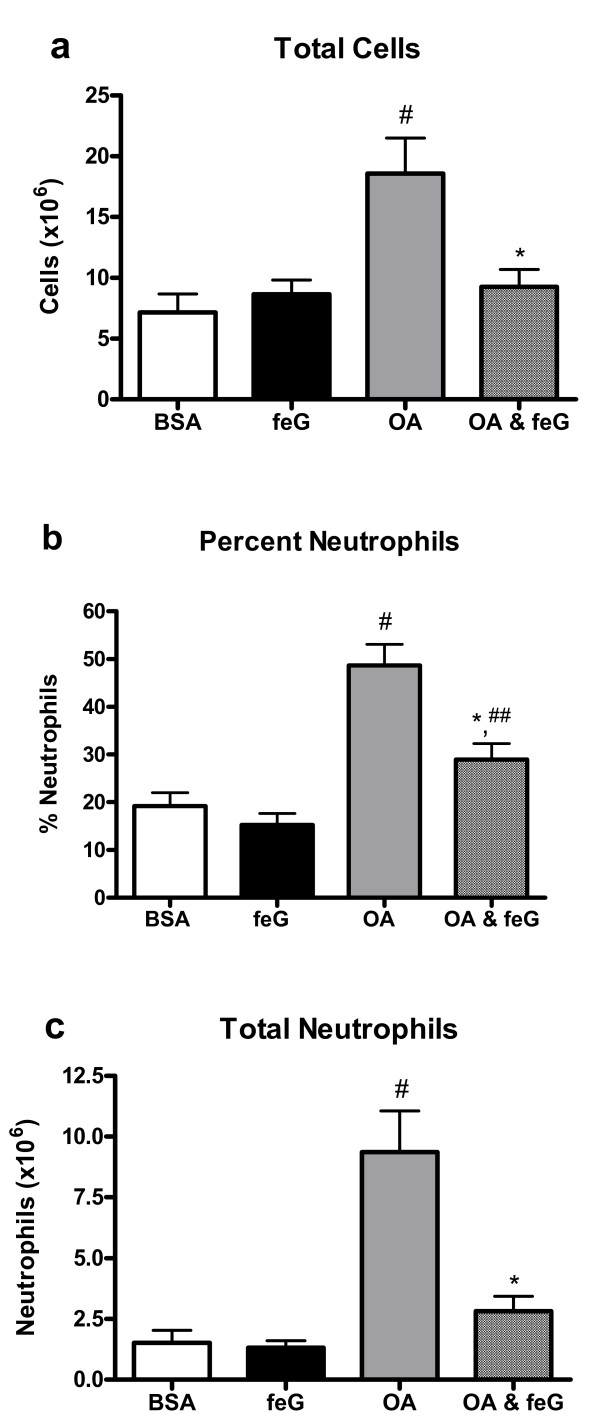
**Leukocyte Counts**. Total leukocyte numbers and the number and percent neutrophils in blood of sensitized rats 18 hours after receiving either naïve antigen (BSA□ n = 9), feG (■ n = 9), sensitizing antigen (OA; n = 11), or OA + feG (; n = 13). Challenge with sensitizing antigen (OA) increased the total number of circulating leukocytes, and this increase was prevented by feG (**a**). Antigen challenge increased significantly the percentage of circulating neutrophils (**b**), which is reflected in a dramatic increase in the total number of circulating neutrophils (**c**). These changes elicited by antigen challenge were inhibited significantly by feG. Significance: # > BSA; ## > feG;* < OA

With unchallenged animals the circulating white blood cell count was 7 ± 2 × 10^6 ^cells/ml, and this number was increased by antigen challenge to 18 ± 3 × 10^6 ^cells/ml (Figure 1a). Treatment with feG, which did not affect neutrophil numbers in unchallenged animals, reduced this antigen-induced increase to 9 ± 1 × 10^6 ^cells/ml. When the percentage of neutrophils is considered a more exaggerated response of antigen challenge was revealed. Between 15 and 19% of the circulating leukocytes examined by FACS analysis were neutrophils in BSA and feG treated animals (Figure 1b). However, 18 h after antigen challenge the percentage of neutrophils in the blood increased 3-fold to 49 ± 4%, which given the doubling of the total number of circulating leukocytes reflects a 6-fold increase in the number of circulating neutrophils (Figure 1c). feG treatment reduced the increase in the percentage of neutrophils to 29 ± 3%, which reflects a decrease of 70% in the total number of circulating neutrophils relative to the OA-challenged animals.

### Intracellular Oxidative Activity

**Figure 2:**
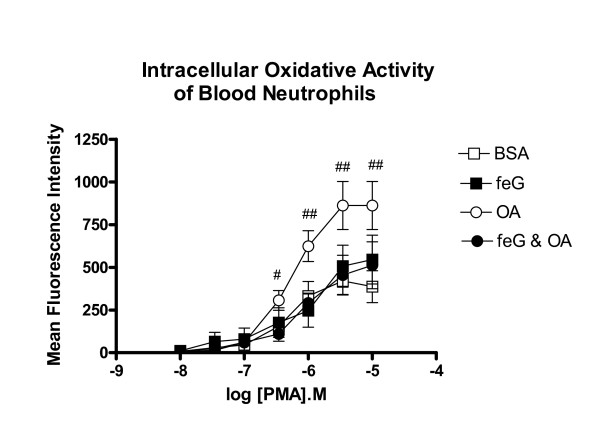
**Intracellular Superoxide**. Dose response for PMA stimulation of intracellular oxidative activity of circulating neutrophils 18 hours after administering to ovalbumin (OA)-sensitized rats naïve bovine serum albumin (BSA) (□ n = 7), sensitizing OA antigen (○, n = 7), feG (■ n = 7), or OA + feG (●, n = 6). Oxidative activity was measured using flow cytometry for a marker of oxygen free radicals (123-dihydrorhodamine), and is expressed as mean fluorescence intensity (MFI). Significance: # < feG & OA; ## > all other groups.

Background fluorescence of the neutrophils in the presence of DHR alone was the same with all animal groups – BSA challenged, feG-treated, OA-challenged, and feG-treated & OA-challenged (not shown). PMA, in the dose range of 3.5 × 10^-7^M to 10^-5^M, increased intracellular ROS production by circulating neutrophils collected from antigen challenge (OA) animals (Figure 2). Treatment with feG at the time of antigen challenge prevented this increase, such that PMA-stimulated ROS production was comparable to that seen with control animals (i.e. BSA-challenged or feG treated).

In several experiments the effects of feG, added to cells *in vitro*, on intracellular oxidative activity were examined. The background for cells obtained from unsensitized rats was 66.2 ± 7.6 MFI and PMA (3.5 × 10^-7^M) increased fluorescence to 142.7 ± 24.9 MFI. feG in the concentration range of 10^-8^M to 10^-13^M modified neither background nor PMA stimulated oxidative activity, with representative values for 10^-11^M feG being 71.1 ± 10.2 and 130.0 ± 16.6 MFI for background and PMA-stimulated cells, respectively.

### Protein Kinase C (PKC) inhibition and intracellular Oxidative Activity

**Figure 3:**
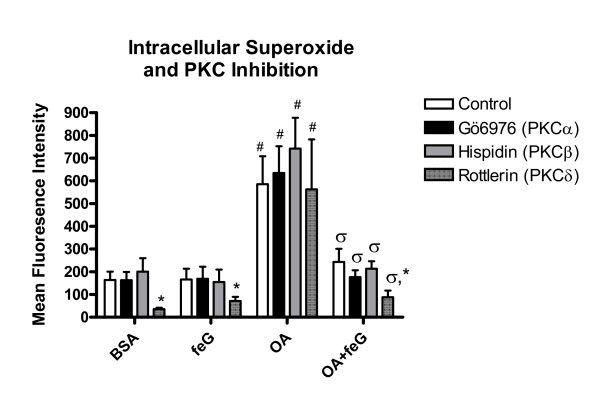
**PKC Inhibition and Intracellular Superoxide**. Effects of several PKC isozyme inhibitors (Control (no PKC inhibitor) □ Gö6976/PKCα ■ hispidin/PKCβ ; and rottlerin/PKCδ) on PMA-stimulated (3.5 × 10^-6^M) ROS production by circulating neutrophils. Oxidative activity of circulating neutrophils 18 hours after administering to sensitized rats either BSA (n = 5); feG (n = 6); OA antigen (n = 6), or OA + feG (n = 6). Oxidative activity was measured by determining mean fluorescence intensity (MFI) using flow cytometry for a marker of oxygen free radicals (123-dihydrorhodamine; DHR). Significance: * < Control; # > BSA; σ < OA

With circulating neutrophils neither the PKCα inhibitor, Gö6976, nor the PKCβ inhibitor, hispidin, altered the generation of PMA-stimulated ROS in any of the animal groups, indicating an independence of ROS production from PKCα and PKCβ (Figure 3). However, with the naïve antigen, BSA, either in the presence or absence of feG, ROS generation by circulating neutrophils was reduced by ~ 70% with the PKCδ inhibitor, rottlerin. This inhibitory effect of rottlerin was abolished after antigen challenge (OA), suggesting that allergic reaction alters the ability of PKCδ to modulate the activation of NADPH oxidase activity in neutrophils. feG restored PKCδ regulation of ROS production after OA-challenge, indicating a modulation of PKCδ activity by the peptide.

### Extracellular release of superoxide anion

**Figure 4:**
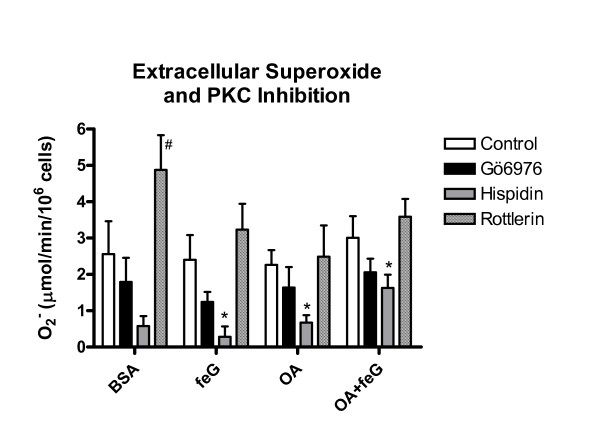
**PKC Inhibition and Superoxide Release**. Effects of PKC isozyme inhibitors (Control □ Gö6976/PKCα ■ hispidin/PKCβ ; and rottlerin/PKCδ) on PMA (3.5 × 10^-6^M)-stimulated superoxide release from circulating neutrophils. Oxidative activity was measured 18 hours after administering to ovalbumin (OA)-sensitized rats naïve bovine serum albumin (BSA) (n = 5), sensitizing OA antigen (n = 6), feG (n = 4), or OA + feG (n = 6). Oxidative activity was measured by determined by reduction of cytochrome C. The results are expressed as μmoles/min/10^6 ^neutrophils. Significance: * < Control; # > Control.

For all groups of animals the PMA-stimulated superoxide anion release from circulating leukocytes of PMA-stimulated (control cells) was similar (Figure 4). The PKCα inhibitor-treated (Gö6976) did not modify PMA-stimulated superoxide anion release from leukocytes, whereas hispidin reduced superoxide release in all animal groups, thus indicating a PKCβ involvement in the extracellular release of superoxide anion. Rottlerin, the PKCδ inhibitor, significantly increased superoxide release from circulating leukocytes of the BSA-challenged animals, although this increase did not occur with the other treatment groups.

### Cell surface expression of CD11b/c and CD49d

**Figure 5:**
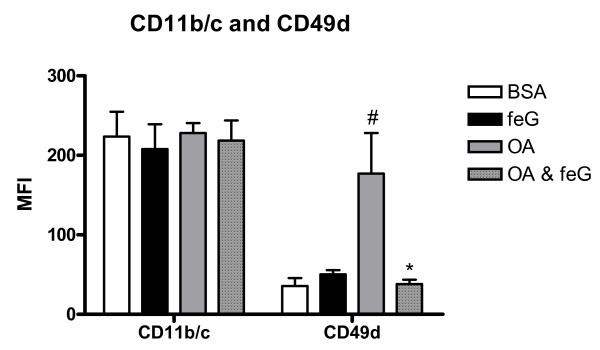
**Cell Surface Expression of CD11b/c and CD49d**. The effect of antigen challenge on the expression of CD11b/c β 2-integrin and CD49d β 1-integrin on circulating neutrophils. Integrin expression on the cell surface of neutrophils was determined by measuring the mean fluorescence intensity (MFI) of specific antibody binding for each integrin. Ovalbumin sensitized rats received either naïve antigen (BSA □ n = 5), feG (■ n = 6), sensitizing antigen (OA ; n = 6), or OA + feG (; n = 6) 18 h before harvesting the cells. Significance: # > BSA; * < OA.

Treatment with feG reduced the antigen challenge-induced increase in expression of CD49d on circulating neutrophils, whereas CD11b/c expression was not affected by any of the treatments (Figure 5).

## Discussion

The respiratory burst of neutrophils functions as a primary host-defence mechanism against invading micro-organisms. This microbicidal action occurs predominately inside the cell within the phagolysosome [23], and normally only a small portion of superoxide or its metabolites is released to the extracellular environment [24,25] through the orifice formed by fusion of oxidant-producing compartments with the plasma membrane [24]. However, the superoxide that is released extracellularly is transformed into H_2_O_2 _with the concurrent release of myeloperoxidase, which reacts with a halogen (e.g. Cl^-^) to form the highly toxic hypochlorous acid (HOCl). It is this extracellular generation of ROS that is believed to contribute to aggravated inflammation and cell damage in several diseases such as systemic inflammatory response syndrome [26], hypoxic injury followed by reoxygenation after transplantation and in myocardial, hepatic, intestinal, cerebral, renal, other ischemic diseases [27], and pulmonary inflammation [28].

The extracellular release of superoxide by circulating neutrophils and eosinophils is increased in patients with asthma [29-32] or cutaneous allergic reactions [33,34]. The results of the current study show that an increase in the respiratory burst of circulating neutrophils also occurs with intestinal allergy, and may be a general feature of type I hypersensitivity reactions, although in our animal model it is predominately the generation of intracellular ROS within neutrophils that is increased by antigen challenge, whereas superoxide release is not altered. Normally, the NADPH oxidase complex in circulating leukocytes is unassembled and functionally inactive, a mechanism that prevents inappropriate generation of superoxide. However, upon exposure to a priming agent the NADPH oxidase complex is assembled so that after extravasating and migrating to the site of inflammation the phagocyte is functionally active [23]. The results described herein suggest that an allergic reaction inappropriately primes the NADPH oxidase complex in circulating neutrophils, and although ideally the superoxide generated is directed into the phagolysosome a small portion of superoxide or its metabolites is released to the extracellular environment [24,35]. This extracellular appearance of neutrophil-derived ROS that contributes to aggravated inflammation and cell damage. Interference with ROS production [36] may account for the therapeutic potential of some anti-asthmatic or anti-allergic drugs [37-39]. Similarly, the anti-allergic and anti-asthmatic properties of feG [6,7] may be due, in part, to the reduction in the intracellular oxidative burst activity of neutrophils.

Several PKC isozymes (α, βII, δ and ζ) are involved in the regulation of NADPH oxidase and the respiratory burst of human and rat neutrophils [40-47], a process that involves phosphorylation by these four PKC isozymes of p47^*phox *^[41,43,47]. This phosphorylation is a critical step for translocation of the cytosolic components and assembly of the active NADPH oxidase. Of particular relevance to PMA-stimulated generation of ROS in neutrophils are the PKC isozymes α, β, and δ. These isozymes require for their activation DAG, the endogenous ligand for PMA, whereas the PKCζ isoform, does not require DAG. Intracellular ROS production by circulating neutrophils is regulated predominately by PKCδ (Figure 3), and this result concords with reported role of PKCδ in regulating NADPH oxidase assembly for PMA-dependent generation of ROS in human neutrophils [48], monocytes [49,50] and eosinophils. Generally, PKCδ is considered to positively regulate superoxide release from human eosinophils [51,52], and the increase in PMA-stimulated release of superoxide from neutrophils of rats challenged with BSA (naïve antigen) in the presence of the PKCδ inhibitor, rottlerin (Figure 4) seems paradoxical. This potentiating action of rottlerin possibly reflects the positive and negative role of PKCδ in regulating cell function, as a similar increase in superoxide release was seen with zymosan-stimulated equine eosinophils [53], although data on neutrophils are lacking. It may be possible that PKCδ participates in shifting the direction of ROS production from intracellular accumulation to extracellular release, although this speculation requires confirmation. Given that eosinophils from atopic patients release superoxide predominately into the extracellular space, whereas that of neutrophils is directed more to the interior of the cell [54], it would be interest to determine if the directional differences reflect the different contributions of PKCδ to the Rac-dependent site of assembly of the NADPH oxidase complex in eosinophils and neutrophils, i.e. plasma membrane or phagolysosome, respectively [54].

In contrast, the release of superoxide from neutrophils is regulated predominately by PKCβ [43,45], an observation that was corroborated in the present study (Figure 4). Our study also shows that antigen challenge of sensitized animals leads to loss of responsiveness to PKC inhibitors, as seen with the PKCδ inhibitor, rottlerin, on circulating neutrophils (Figure 3). This loss of responsiveness to rottlerin may reflect a deregulation of PKC by antigen challenge. The mechanism by which this occurs is not known, but may reflect a recently described novel G-protein receptor coupled (GPCR)-PKC-regulated switch that enhances receptor signalling, and prevents receptor internalization with consequent loss of responsiveness [55]. Treatment with feG re-established sensitivity to rottlerin, and corrected the supposedly deregulated PKC function, although the mechanism of action is unknown.

An up-regulation of CD49d expression on circulating neutrophils occurs with ischemia-reperfusion injury [56], in septic patients [57], and as shown herein with allergic reactions (Figure 5). This abnormal up-regulation of a β1-integrin on circulating neutrophils leads to inappropriate neutrophil homing and recruitment [56-58], and activation of NADPH oxidase [59,60]. Thus, expression of β1-integrin on circulating neutrophils could cause inappropriate inflammatory responses not only at the leukocyte-endothelial cell interface but also at an extravascular interface [9,59], possibly through a mechanism involving frustrated phagocytosis and the leakage of the dismutated product of intracellular superoxide, hydrogen peroxide, from intracellular compartments. Concurrent with a decreased expression of CD49d by feG treatment of OA-challenged animals (Figure 5) the intracellular oxidative burst was correspondingly decreased (Figure 3) with a consequent reduction in the severity of allergic reactions. These observations may explain why antibodies to and small molecule antagonists against CD49d are effective in blocking asthmatic reactions in rats and sheep [61,62].

The mechanism by which feG, administered 18 h after antigen, decreases circulating neutrophil accumulation, intracellular oxidative activity and CD49d expression remains undefined. However, previous studies suggest that feG and related peptides probably exert their anti-allergic actions on early cellular events as they reduce rapidly initiated anaphylactic events such as hypotension, intestinal motility and vascular permeability [10,20]. A mode of action for feG independent of mast cells may predominant as the peptides do not modify antigen-evoked mast cell degranulation [4], whereas this peptide effectively reduces neutrophil adhesion and leukocyte migration both *in vivo *and *in vitro *[6,17]. Since neither binding nor cellular uptake of [^3^H]feG has been observed with rat leukocytes or neutrophilic transformed HL60 cells (unpublished), we are currently determining if feG may act as a high affinity, low avidity allosteric regulator of integrins and associated co-stimulatory molecules [17], in a manner similar to a regulation of CD11a/CD18 affinity for counter ligands by a conformational switch in the I domain of this integrin [63]. Since engagement of integrins contributes to increases in vascular permeability and superoxide production [64,65], this mechanism of action may account for the observed properties of feG.

## Conclusion

The tripeptide feG reduces the increased expression of CD49d and intracellular oxidative burst of circulating neutrophils elicited by antigen challenge. feG prevents the loss of responsiveness in the regulation of PKCδ in circulating neutrophils.

## Abbreviations

BSA: bovine serum albumin; DHR: dihydrorhodamine 123; FACS; Fluorescence-Activated Cell Sorter; feG: D-phenylalanine-D-glutamate-glycine; FEG: L-phenylalanine-L-glutamate-glycine; MFI: mean fluorescence intensity; OA: ovalbumin; PAF: platelet-activating factor; PKC: protein kinase C; PMA: phorbol myristate acetate; PMN: polymorphonuclear leukocyte (PMN); ROS: reactive oxygen species

## Competing interests

The author(s) declare that they have no competing interests.

## Authors' contributions

All authors participated in study design and read and approved the final manuscript. JSD aided in protocol development and critically reviewed the manuscript. RM coordinated the study, analyzed the data with statistical analysis and prepared the manuscript.
